# Evolutionary mechanisms underpinning fitness response to multiple stressors in *Daphnia*

**DOI:** 10.1111/eva.13258

**Published:** 2021-06-10

**Authors:** Maria Cuenca‐Cambronero, Jelena H. Pantel, Hollie Marshall, Tien T. T. Nguyen, Henar Tomero‐Sanz, Luisa Orsini

**Affiliations:** ^1^ Department of Fish Ecology and Evolution Eawag Kastanienbaum Switzerland; ^2^ Aquatic Ecology and Evolution Institute of Ecology and Evolution University of Bern Bern Switzerland; ^3^ Environmental Genomics Group, School of Biosciences University of Birmingham Birmingham UK; ^4^ Department of Computer Science, Mathematics, and Environmental Science The American University of Paris Paris France; ^5^ Institute of Evolutionary Biology University of Edinburgh Edinburgh UK; ^6^ Department of Animal and Plant Sciences University of Sheffield Sheffield UK; ^7^ Department of Molecular Cancer Pathology of IRYCIS Hospital Ramon y Cajal Madrid Spain

**Keywords:** additive, antagonistic, biocides, evolution, multistress, plasticity, resource availability, resurrection ecology, synergistic

## Abstract

Multiple stressors linked to anthropogenic activities can influence how organisms adapt and evolve. So far, a consensus on how multiple stressors drive adaptive trajectories in natural populations has not been reached. Some meta‐analysis reports show predominance of additive effects of stressors on ecological endpoints (e.g., fecundity, mortality), whereas others show synergistic effects more frequently. Moreover, it is unclear what mechanisms of adaptation underpin responses to complex environments. Here, we use populations of *Daphnia magna* resurrected from different times in the past to investigate mechanisms of adaptation to multiple stressors and to understand how historical exposure to environmental stress shapes adaptive responses of modern populations. Using common garden experiments on resurrected modern and historical populations, we investigate (1) whether exposure to one stress results in higher tolerance to a second stressor; (2) the mechanisms of adaptation underpinning long‐term evolution to multistress (genetic evolution, plasticity, evolution of plasticity); and (3) the interaction effects of multiple stressors on fitness (synergism, antagonism, additivity). We measure the combined impact of different levels of resource availability (algae) and biocides on fitness‐linked life‐history traits and interpret these results in light of historical environmental exposures. We show that exposure to one stressor can alter tolerance to second stressors and that the interaction effect depends on the severity of either stressor. We also show that mechanisms of adaptation underpinning phenotypic evolution significantly differ in single‐stress and multistress scenarios. These adaptive responses are driven largely by synergistic effects on fecundity and size at maturity, and additive effects on age at maturity. Exposure to multiple stressors shifts the trade‐offs among fitness‐linked life‐history traits, with a stronger effect on *Daphnia* populations when low‐resource availability and high biocide levels are experienced. Our study indicates that mitigation interventions based on single‐stress analysis may not capture realistic threats.

## 1 INTRODUCTION

Environmental change driven by human activities exposes wildlife to multiple stressors, leading to biodiversity loss up to 1000 times the natural rate (Brook et al., 2008; Hallmann et al., 2017). Chemical pollution is one of the major global threats to biodiversity; biocides are among the substances of concern for regulators and policymakers for their documented adverse effect on humans and the environment and their potential bioaccumulation properties (Brook et al., 2008; García‐Vega & Newbold, 2020; Häder et al., 2020). Biocides are the main source of surface water pollution (Moss, 2007). A number of studies on environmentally relevant concentrations of common‐use biocides (ng/L to µg/L) have documented adverse effect on aquatic organism endpoints, including sex determination, fecundity, adult size, and mortality (e.g., Kashian & Dodson, 2002). Despite documented adverse effects of biocides on freshwater species, cases of increased baseline tolerance (genetic adaptation) and plasticity have been documented (Coors et al., 2009; Cuenca‐Cambronero et al., 2018; Jansen et al., 2015).

Many studies to date have focused on the impact of biocides in the absence of other components of environmental degradation. However, a multistress perspective is needed to grasp the full impact of biocides on natural ecosystems, reflecting the complexity of natural habitats (Sinha et al., 2017). This is because the combined effect of multiple stressors can result in additive (equal to the sum of single stressors), antagonistic (less than the sum of single stressors), or synergistic (greater than the sum of single stressors) effects on biological endpoints (e.g., mortality) and fitness traits (e.g., fecundity). The prevalence of these combinations varies with the level of biological organization (populations, species, and communities) and also among species (Bracewell et al., 2019). Meta‐analyses on the impact of multiple stressors on community composition across different habitat types report more frequently additive and then synergistic effects (Cote et al., 2016). In freshwater ecosystems, antagonistic effects are more common (41%) than synergistic (28%) or additive (16%) interactions; these statistics are drawn from >230 studies encompassing experimental populations and field observations, and response variables ranging from survival and growth to biomass and diversity (Jackson et al., 2016). Species‐level analysis identifies synergism in at least 50% and up to 68% of studies (Holmstrup et al., 2010; Schafer & Piggott, 2018). The outcome of these effects depends on the stressors investigated (e.g., pathogens and heat stress combined with biocides) (Holmstrup et al., 2010). Biocide impact on freshwater species has been shown to be more severe in presence of predation (Jansen, Coors, et al., 2011), possibly explained by changes in energy storage and metabolism required in predator avoidance mechanisms (Beckerman et al., 2007; Oda et al., 2019). Resource availability can change the tolerance level to biocides; higher resource availability enables higher baseline tolerance than limiting resource availability, indicating that adaptive potential of species is not only stressor‐dependent but also influenced by the interaction among multiple stressors (Wuerthner et al., 2019). For example, algal resource availability and insecticides show antagonistic effects on invertebrates in microcosm experiments, impacting abundance, biomass, richness, size, structure, and composition of the community (Chara‐Serna & Richardson, 2018).

The number of studies on multiple stress exposure is increasing, providing us with a better understanding of the fitness costs associated with exposure to complex environments (e.g., Cuenca‐Cambronero et al., 2018; Cuenca‐Cambronero et al., 2018; Jansen, De Meester, et al., 2011; Wuerthner et al., 2019; Zhang et al., 2019). However, we do not know how past exposure to multiple stressors influences response mechanisms to modern environments (but see Cuenca‐Cambronero et al., 2018; Cuenca‐Cambronero et al., 2018) and how multiple stressors may influence evolutionary responses through time. It is unlikely that all stressor combinations can be experimentally tested. However, quantifying the effects of combinations that are common in the landscape may identify generalities about ecosystems, stressors, and responses, using similarity of chemical structure (e.g., biocides) and toxicological properties (e.g., mode of action of chemicals on molecular pathways) of stressors and providing means to reliably predict interactions between novel combinations (Bracewell et al., 2019; Cote et al., 2016).

The keystone species *Daphnia* is a tractable invertebrate model: They are easy and inexpensive to culture in the laboratory, have a short life cycle, and produce large numbers of externally laid embryos. *Daphnia* has a parthenogenetic life cycle, in which sexual and asexual reproduction alternate (Ebert, 2005). Sexual recombination results in early‐stage embryos that arrest their development and enter dormancy (Kerfoot & Weider, 2004). Dormant embryos can be revived from different times in the past providing the opportunity to study long‐term adaptive responses to environmental change. Resurrected embryos can be propagated clonally under standard laboratory conditions, allowing the rearing of populations of isogenic individuals (clones) from a single genotype (Cuenca‐Cambronero & Orsini, 2018).

Here, we study three populations of *Daphnia magna* resurrected from a sedimentary archive of Lake Ring, Denmark (55°57′51.83″N, 9°35′46.87″E) (Cuenca‐Cambronero et al., 2018). The lake has a well‐documented history of human impact: In the late 1950s, it experienced eutrophication due to sewage inflow from a nearby town, which was diverted in the late 1970s; from the 1980s until the late 1990s, the lake experienced an increase in biocide run‐off due to agricultural land use intensification and high nutrient levels; the lake partially recovered from high nutrient levels and biocides in modern times (>1999s) (Cuenca‐Cambronero & Orsini, 2018; Cuenca‐Cambronero et al., 2018). According to the Danish county authority, carbamate insecticides were among the top sold biocides between the 1980s and 1990s (Cuenca‐Cambronero et al., 2018; www.middeldatabasen.dk). Although biocide persistence in sediment may vary greatly among compounds and with redox conditions, carbamates are very mobile and virtually nondegradable under anaerobic conditions; therefore, they are highly persistent in sediment and soil (Bondarenko & Gan, 2004). Given the lake historical background, the *Daphnia* populations experienced exposures to stress mixtures over time, specifically involving resource availability and biocide levels. The *Daphnia* populations experienced the following environments: (1) the eutrophic population (EP) experienced high food levels and no biocides, (2) the pesticide population (PP) experienced high food availability and high biocide exposures, and (3) the clear‐water phase population (CWP) experienced low food availability and low biocide exposure.

We study the combined effect of different levels of biocides, using the carbamate insecticide Carbaryl as proxy, and of resources availability (algae) on the resurrected populations of *D*. *magna*. We use the carbamate insecticide Carbaryl (Pestanatal) at concentrations previously shown to have adverse effects on *Daphnia* fitness, impacting survival and fecundity (e.g., Cuenca‐Cambronero et al., 2018; Jansen, Coors, et al., 2011). Our study addresses three main questions:

1. Does exposure to one stressor result in higher tolerance to a second stressor?
2. What mechanisms of adaptation (genetic evolution, plasticity, evolution of plasticity) enable long‐term evolution to multistress environments?
3. Is synergism more common than other interactions in *Daphnia*, supporting evidence from other population‐level studies?

The detailed knowledge of the historical environment of the temporal populations and their origin from the same genetic pool provides an optimal system to understand the evolutionary mechanisms underpinning adaptation to complex environments. By comparing historical and modern populations in different environments, we are able to investigate mechanisms of adaptation and tolerance to multiple stress and to understand how historical exposure to certain environmental stressors shapes adaptive responses of modern populations. Using an additive null model (Piggott et al., 2015), we quantify antagonistic, synergistic, and additive interactions of two insecticide levels and two food regimes and interpret those in light of the mechanisms shaping trait evolution. We study trade‐offs among fitness‐linked life‐history traits driven by single stressors and pairs of stressors. Our study identifies the mechanisms that enable populations' adaptive responses to complex environments.

## 2 MATERIAL AND METHODS

### 2.1 Experimental design and study system

**FIGURE 1:**
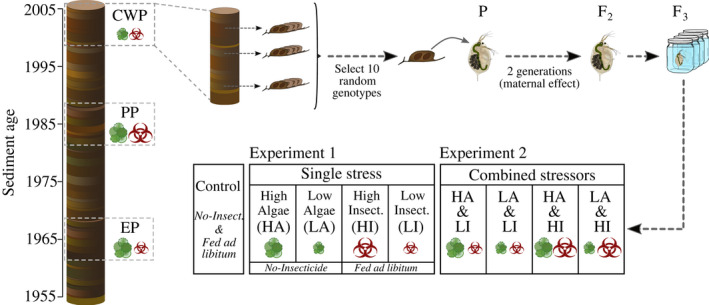
Experimental design and study system. Populations separated in time of *Daphnia magna* were revived from dormant embryos resurrected from a sedimentary archive of Lake Ring (Denmark). From each population, 10 genotypes were used in the experiments presented here (*N* = 30). The three time periods are as follows: (1) eutrophication (EP)—1960–1970—high nutrients and low/no concentration of biocides, (2) pesticide (PP)—1980–1990—high concentration of nutrients and biocides, and (3) clear‐water (CWP) ‐ >1999—low concentration of nutrients and biocides. The 30 genotypes were kept in standard laboratory conditions for two generations (16:8‐h light: dark regime, 20°C and fed *ad libitum* with 0.8 mg carbon/L of *Chlorella vulgaris* daily) to control for maternal effect. Following the acclimation phase, clonal replicates of the 30 genotypes were used in multiple common garden experiments in which two algae levels (0.2 mg C/L and 2.4 mg C/L) and two insecticide Carbaryl concentrations (4 and 8 μg/L) were tested in isolation and factorial combinations on a suite of fitness‐linked life‐history traits. HA, high algae; HI, high insecticide; LA, low algae; LI, low insecticide

*Daphnia magna* dormant embryos were revived from a sedimentary archive of Lake Ring, Denmark (55°57′51.83″N, 9°35′46.87″E) (Cuenca‐Cambronero & Orsini, 2018; Cuenca‐Cambronero et al., 2018). Lake Ring has a well‐known history of human impacts and experienced three main phases: eutrophication (EP)—1960–1970—high concentration of nutrients and low/no concentration of biocides, pesticide (PP)—1980–1990—high concentration of nutrients and biocides, and clear‐water (CWP)—>1999—low concentration of nutrients and biocides (Cuenca‐Cambronero & Orsini, 2018; Cuenca‐Cambronero et al., 2018). *Daphnia magna* were isolated and hatched from each lake phase (hereafter referred to as populations EP, PP, and CWP) and maintained as isoclonal lines in standard laboratory conditions for several generations (16:8‐h light: dark regime, 10°C and 0.4 mg carbon/L of *Chlorella vulgaris* biweekly). From each time period, ten distinct genotypes were randomly selected to represent the population from that time to be used in this study (Figure 1). The sample size per population was chosen based on previous results showing that 10 genotypes are representative of the local genetic diversity (Orsini et al., 2016). At the start of the experiment, the 30 genotypes were transferred to the following conditions for at least two generations to reduce interference from maternal effect and synchronize reproduction: 16:8‐h light: dark regime, 20°C, and fed *ad libitum* with 0.8 mg carbon/L of *Chlorella vulgaris* daily; the growth medium (borehole water, collected from a deep well with stable microbiological composition and protected from rainwater) was renewed three times per week. After purging (grand)maternal effects, clonal replicates of the 30 genotypes were placed in common garden experiments to measure the effects of single and combined stressors on fitness. Due to large number of genotypes and conditions tested, exposures were run in three batches. Juveniles of 24–48 h from the second or following broods of the second generation were individually exposed to two food (algae) levels (0.2 mg C/L and 2.4 mg C/L of *C*. *vulgaris*) and two insecticide Carbaryl (Pestanatal) concentrations (4 and 8 µg/L), as well as to combinations of algae and Carbaryl at the two tested levels (Figure 1). A control group was used throughout the exposures for posterior normalization of data and consisted of clonal lines of the same 30 genotypes kept in stress‐free conditions (i.e., no Carbaryl and *fed ad libitum* 0.8 mg C/L of *C*. *vulgaris* daily). Fitness‐linked life‐history traits were measured in the single and multiple stressor common garden experiments for the duration of each individual's life cycle (i.e., until all experimental animals released their second brood). The life‐history traits measured were as follows: (1) fecundity, quantified as total number of offspring in first and second brood; (2) size at maturity, measured as the distance between the head and the base of the tail of adult *Daphnia* (mm); (3) age at maturity, age of release of first brood in the brood pouch (days); and (4) mortality, recorded as the day at which an animal went extinct in the course of the experiment spanning an individual's life cycle. Experimental animals were observed daily.

A first goal of our experiments was to quantify the impacts of each stressor in the absence of the other stressor and then to measure the combined effect of multiple stressors. For this reason, we ran two separate common garden experiments. In the single‐stressor experiment, we expose the three populations of *D*. *magna* to each of four environments: high algae (HA; 2.4 mg C/L), low algae (LA; 0.2 mg C/L), high insecticide (HI; 8 µg/L), and low insecticide (LI; 4 µg/L and). In the single‐stressor experiments with varying algae level, there was no Carbaryl present while in the single‐stressor experiments with varying Carbaryl level, animals were fed ad libitum (the same algae level used during the phase of purging of maternal effect; 0.8 mg carbon/L of *Chlorella vulgaris* per day). This feeding regime differs from the low algae level (LA) and the saturated algae levels (HA) treatments in the food regime experiments. In the combined‐stressor experiment, we expose the three populations of *D*. *magna* to a factorial combination of high and low algae and high and low Carbaryl levels—treatments were thus high algae combined with high and low insecticide (HAHI and HALI, respectively) and low algae combined with high and low insecticide (LAHI and LALI, respectively) (Figure 1).

### 2.2 Life‐history and fitness response to single and multiple stressors

Prior to the statistical analyses, the three batches of experiments were standardized by subtracting the standard deviation of the controls from the observed values (*y_i_* ‐ *σ*_control_). For the single‐stressor experiment, we assessed the total variance for three of the life‐history traits (fecundity, size at maturity, and age at maturity), using two separate two‐way ANOVAs, that is, to test for the effects of population and each stressor separately (*y* ~ population * algae and *y* ~ population * insecticide). For the combined‐stressor experiment, we used a three‐way ANOVA, followed by a post hoc analysis, to test for the effect of population and combined stressors (*y* ~ population * algae * insecticide). We applied a linear mixed model (LMM) with genotype as a random effect nested within populations, using a Gaussian distribution for the continuous dependent variables (fecundity, size at maturity, and age at maturity). To assess population and treatment effects on mortality, we fitted a parametric survival model where the day of mortality and the mortality event (dead/alive) combined were treated as dependent variables (following a binomial distribution) and population, algae, and insecticide were used as fixed terms (using the R package “*rms*”; R Core Team, 2019). Similar to the analyses for the other life‐history traits, we applied separate analyses for the single‐stressor experiments and for the combined‐stressor experiment (see above). All statistical analyses were performed using R version 3.6.1 (R Core Team, 2019).

### 2.3 Mechanisms of adaptation to changing stress levels

The analyses described in the previous section provide information on the total trait variation observed in the experiments. A second goal of our experiments was to quantify the mechanisms that structured trait changes in the transitions between lake phases (i.e., EP → PP and PP → CWP). These transitions and the respective environments are visualized in Figure S1. We used the data from both the single‐ and the combined‐stressor experiments to address this question, focusing on environmental conditions that reflect the populations' historical environments. To quantify the impacts of population and environment for observed trait shifts, we used ANOVAs with planned contrasts to analyze variation in phenotypes going from the population and environment that was more distant in time to the population and environment that are more recent in time. In the first transition (T1 → T2), EP was set as 0 and PP as 1. In this transition, environment HALI was set as 0 and HAHI was set as 1. In the second transition (T2 → T3), PP was set as 0 and CWP as 1; environment HAHI was set as 0 and LALI as 1 (Figure S1). In addition to the multistress scenarios, we quantify changes in hypothetical single‐stress scenarios: changes from LI to HI (set as 0 and 1, respectively) in the first transition (T1 → T2) and changes in algae and insecticide in the second transition (T2 → T3) (from HA, set as 0, to LA, set as 1, and from HI, set as 0, to LI, set as 1). We include the historical (combined stressors) and hypothetical (single stressors) environments to illustrate the outcome of single and multistress scenarios.

The resulting effects of population, environment, and their interaction are described in the ANOVAs as: (1) *plasticity* (the change in the mean trait value ($\bar{y}$) in a population between two environments representative of changes between time periods: ${\bar{y}}_{T2}^{\text{EP}}‐{\bar{y}}_{T1}^{\text{EP}}$); (2) *genetic* *evolution* (the difference in mean trait value ($\bar{y}$) between two temporal populations exposed to the same environments: ${\bar{y}}_{T1}^{\text{PP}}‐{\bar{y}}_{T1}^{\text{EP}}$); and (3) *evolution of plasticity* (the difference in mean trait value ($\bar{y}$) between the two environments and temporal populations: $\left({\bar{y}}_{T2}^{\text{PP}}‐{\bar{y}}_{T1}^{\text{PP}}\right)‐\left({\bar{y}}_{T2}^{\text{EP}}‐{\bar{y}}_{T1}^{\text{EP}}\right)$) (Figure S1) (Scheiner, 1993; Stoks et al., 2016).

### 2.4 Multiple stressors effects

To test whether the combined effect of pair of stressors tested in the common garden experiments had synergistic, antagonistic, or additive effects, we used a null model of additivity (Cote et al., 2016), which compares the predicted additive effect of two stressors (i.e., the sum of each individual stressor) with the observed effect of both stressors in isolation. We are able to make these inferences because we exposed the same set of genotypes to both individual stressors and combinations thereof. The null addictive prediction of the joint effect of pairs of stressors in the experiments is calculated as: *E*_mix_ = *E*_A_ + *E*_B_ where *E*_mix_ represents the effect of the joint stressors and E_A_ and E_B_ are the effects of individual stressors (i.e., the effects measures in the single‐stressor experiment; Crain et al., 2008; Piggott et al., 2015; Robin et al., 2017). For these calculations, we used standardized effect sizes, that is, for each trait *y*, we used (${\bar{y}}_{\text{treatment}}‐{\bar{y}}_{\text{control}}$)/*σ*, where *σ* gives the shared standard deviation of the pooled control and treatment trait values (Cote et al., 2016). The effect of combined stressors was considered additive if the predicted joint effect *E*_mix_ was within the 95% confidence intervals of the observed effect from the multistressor experiment (HAHI, HALI, LAHI, LALI), and, thus, there was no significant difference between observed and predicted effects. Interactions were considered antagonistic if the predicted joint effect was smaller and synergistic if the predicted joint effect was larger than the 95% confidence intervals. We used a *t*‐test to assess deviations from the additive null model.

We assessed trade‐offs among size at maturity, age at maturity, and fecundity using ternary plots that show the ratio of each trait relative to two others. Life‐history trait raw data (i.e., fecundity, size at maturity, and age at maturity) were scaled to mean 0, standard deviation 1, then placed into their position in 100 quantile groups or bins. To visualize the overall change in life‐history traits across all experimental conditions, the mean position of each trait and population in each experimental condition were plotted [i.e., from the single‐stressor experiments (HA, LA, HI, and LI) and from the multistressor experiments (HAHI, HALI, LAHI, and LALI)]. The ternary plots were generated using the R package *Ternary* v1.2.0 (Smith, 2017).

## 3 RESULTS

### 3.1 Life‐history and fitness response to single and multiple stressors

**FIGURE 2:**
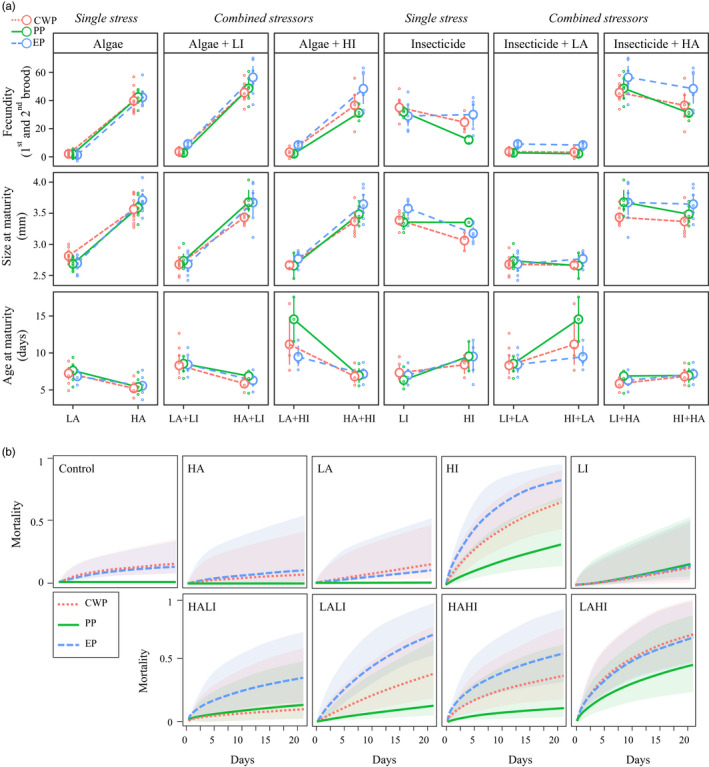
Fitness response to single and multiple stressors. (a) Univariate reaction norms showing response of fitness‐linked life‐history traits (fecundity, size, and age at maturity) to single stressors (insecticide, algae) and combinations of stressors in the populations of *D*. *magna* used in the common garden experiments. The single‐stressor experiments are as follows: HA: high algae (2.4 mg C/L of *C*.* vulgaris*); LA: low algae (0.2 mg C/L of *C*.* vulgaris*); LI: low insecticide (4 μg/L of the carbamate insecticide Carbaryl); HI: high insecticide (8 μg/L of the carbamate insecticide Carbaryl). The multistress experiments are as follows: high algae combined with high and low insecticide (HAHI and HALI, respectively) and low algae combined with high and low insecticide (LAHI and LALI, respectively). (b) Mortality plots with confidence intervals per population are shown for single stressors and combinations of stressors as in point A. Populations are color‐coded: blue/dashed line—EP; green/solid line PP; red/dotted line—CWP. Supporting statistics are in Table 1

We studied the comparative effects of single and multiple stressors on *Daphnia* life‐history traits using two algae levels and two insecticide concentrations. In the single‐stressor experiments involving algae levels (HA and LA), the only significant interaction effect was observed in size at maturity, where populations interacted with algae ($\chi_{2}^{2}$ = 9.454, *p* = 0.009; Table 1). In addition to this effect, fecundity, size at maturity, and age at maturity showed a significant plastic response (fecundity: $\chi_{1}^{2}$ = 1027.828, *p* < 0.001; size at maturity: $\chi_{1}^{2}$ = 603.773, *p* < 0.001; age at maturity: $\chi_{1}^{2}$ = 94.404, *p* < 0.001; Table 1). Low‐resource availability (LA) produced lower fecundity, smaller size at maturity, and a delay in maturation as compared to high‐resource availability (Figure 2a—first column). There was no significant effect of population and resource availability (algae level) on mortality (Table 1).

**TABLE 1:** Univariate ANOVA

	Fecundity (1st and 2nd brood)			Size at maturity (mm)			Age at maturity (Days)			Mortality		
	$\chi^{2}$	*df*	*p*‐value	$\chi^{2}$	*df*	*p*‐value	$\chi^{2}$	*df*	*p*‐value	$\chi^{2}$	*df*	*p*‐value
2‐way ANOVA												
Population	0.693	2	0.707	1.520	2	0.468	0.481	2	2.521	3.842	2	0.147
Algae	1027.828	1	**0.000**	603.773	1	**0.000**	94.404	1	**0.000**	0.225	1	0.636
Pop*Algae	1.410	2	0.494	9.454	2	**0.009**	3.860	2	0.145	0.155	2	0.925
Population	1.856	2	0.395	6.838	2	**0.033**	0.430	2	0.806	3.083	2	0.214
Insecticide	4.894	1	**0.027**	44.285	1	**0.000**	11.202	1	**0.001**	11.430	1	**0.001**
Pop*Insecticide	5.677	2	0.059	5.300	2	0.071	1.807	2	0.405	1.431	2	0.489
3‐way ANOVA												
Population	21.252	2	**0.000**	6.589	2	**0.037**	3.979	2	0.137	9.634	2	**0.008**
Algae	493.042	1	**0.000**	420.573	1	**0.000**	58.522	1	**0.000**	7.574	1	**0.006**
Insecticide	12.094	1	**0.001**	0.361	1	0.548	17.599	1	**0.000**	3.569	1	0.059
Pop*Algae	2.280	2	0.320	5.155	2	0.076	3.109	2	0.211	0.216	2	0.898
Pop*Insecticide	1.452	2	0.484	1.893	2	0.388	2.206	2	0.332	1.824	2	0.402
Algae*Insecticide	7.591	1	**0.006**	1.617	1	0.203	8.274	1	**0.004**	0.159	1	0.690
Pop*Algae*Insect	1.072	2	0.585	0.158	2	0.924	7.747	2	**0.021**	1.646	2	0.439

Note

Univariate analysis of variance (ANOVA) testing the effect of population (P), treatment (algae or insecticide or a combination of both), and their interaction terms (T*P) on fecundity, size at maturity, age at maturity, and mortality for single stressors (2‐way ANOVA) and combination of stressors (3‐way ANOVA). Significant *p*‐values are in bold. The statistics in this table support plots in Figure 2.

In the single‐stressor experiment involving the insecticide Carbaryl, all life‐history traits showed a significant plastic response to the treatment (Table 1). Higher levels of insecticide generally resulted in lower fecundity, smaller size at maturity, delayed maturation (Figure 2a – fourth column), and higher mortality. Populations significantly differed in their size at maturity ($\chi_{2}^{2}$ = 6.838, *p* = 0.033; Table 1).

The effects of multiple stressors on fitness‐linked life‐history traits differed from single‐stressor effects. The three‐way interaction Population * Algae * Insecticide was significant for age at maturity ($\chi_{2}^{2}$ = 7.747, *p* = 0.021; Table 1). While no significant interaction between population and individual treatment was observed for any trait (Table 1), significant plastic response was observed for the combination of algae and insecticide, which had significant effects on fecundity and age at maturity (fecundity: $\chi_{1}^{2}$ = 7.591, *p* = 0.006; age at maturity: $\chi_{1}^{2}$ = 8.274, *p* = 0.004; Table 1). Fecundity ($\chi_{2}^{2}$ = 21.252, *p* < 0.001), size at maturity ($\chi_{2}^{2}$ = 6.589, *p* = 0.037), and mortality ($\chi_{2}^{2}$ = 9.634, *p* = 0.008) significantly differed among populations. The post hoc analysis revealed that the EP population showed a significant increase in fecundity, whereas the CWP population showed a significant smaller size as compared to the other two populations (Table S3, Figure 2a). Mortality was generally higher in multistress than in single‐stress exposures (Figure 2b). The combined stress induced a population‐dependent mortality (Figure 2b). Across experiments, the EP population suffered, on average, higher mortality than PP and CWP (Figure 2b, Table S3).

Our experimental design enabled us to observe the dosage effect of each stressor at two different levels of a second stressor. For example, we could compare the effect of high and low algae at both low‐ and high‐insecticide concentrations. Looking at our results in this perspective, resource availability (algae levels) significantly affected all traits both in low (LI) and high (HI) insecticide levels (Figure 2a; second and third columns). Conversely, different concentrations of insecticide significantly affected age at maturity in low algae (LA) and fecundity in high algae (HA) (fecundity: $\chi_{1}^{2}$ = 12.094, *p* = 0.001; age at maturity: $\chi_{1}^{2}$ = 17.599, *p* = 0.001; Table 1; Figure 2a, fifth and sixth columns).

### 3.2 Mechanisms of adaptation to changing stress levels

We investigated the mechanisms of adaptation [plasticity (E), genetic evolution (G), and evolution of plasticity (G * E)] that enabled the temporal populations of Lake Ring to persist through environmental transitions historically observed in Lake Ring.

**FIGURE 3:**
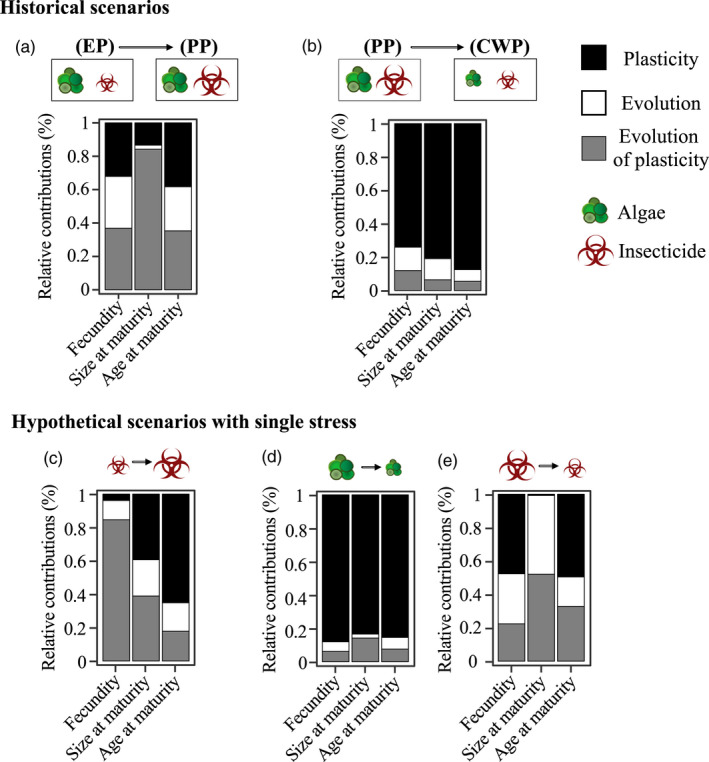
Evolutionary mechanisms in complex environments. Relative contribution of plasticity (black), evolution of plasticity (gray), and genetic evolution (white) in three life‐history traits (fecundity, size at maturity, and age at maturity) from the two transition periods in Lake Ring: (a) eutrophication (EP) to pesticide (PP), and (b) pesticide (PP) to clear‐water (CWP). Panels c, d, e show the relative contribution of plasticity, evolution of plasticity, and genetic evolution when hypothetical single‐stressor scenarios are considered in the EP to PP transition (c) and in the PP to CWP transition (d, e)

In the first transition (T1 → T2, HALI → HAHI), biocides increased, whereas food levels remained unchanged and high—we thus evaluated the effects of change in population from EP to PP and the change in environment from LI to HI under high‐resource availability (Figure 1a, Figure S1). In this transition, plasticity, evolution of plasticity, and genetic evolution contributed equally to changes in fecundity and age at maturity; evolution of plasticity largely contributed (>80%) to changes in size at maturity (Figure 3a); genetic evolution ($\chi_{1}^{2}$ = 7.201, *p* = 0.014) and plasticity ($\chi_{1}^{2}$ = 7.521, *p* = 0.013) significantly affected fecundity. However, coefficient estimates were not significant for size and age at maturity (Table S1).

In the second transition (T2 → T3, HAHI → LALI), both nutrient levels and biocides decreased over time. Thus, we contrasted environments in which both resource availability and biocides decreased. In this transition, only plasticity explained changes in life‐history traits (Figure 3b, Table S1).

We also quantified the relative contribution of the three mechanisms of adaptation [plasticity (E), genetic evolution (G), and evolution of plasticity (G * E)] in the two transitions focusing on single‐stress scenarios. The calculated impact on traits was different when we used data from the single‐stress scenarios, as compared to the multistress scenarios that better capture the historical environment of the studied populations. In the first transition from low to high biocides (EP → PP; LI → HI), evolution of plasticity significantly affected changes in fecundity and size at maturity (fecundity, *p* = 0.04; size at maturity, *p *= 0.047), whereas plasticity significantly drove changes in age at maturity (*p* = 0.008) (Figure 3c, Table S1). In the second transition, we quantified changes in resource availability (algae) (PP → CWP; HA → LA) (Figure 3d) as well as changes in biocide concentration (PP → CWP; HI → LI) in isolation (Figure 3e). In the transition between HA → LA, all changes in fitness‐linked life‐history traits were underpinned by plasticity (Figure 3d, Table S1). In the transition from high to low biocides (Figure 3e, Table S1), changes in fecundity and age of maturity were driven almost equally by genetic evolution, plasticity, and evolution of plasticity, whereas changes in size at maturity were driven by evolution and evolution of plasticity (Figure 3e).

### 3.3 Multiple stressors effects

**FIGURE 4:**
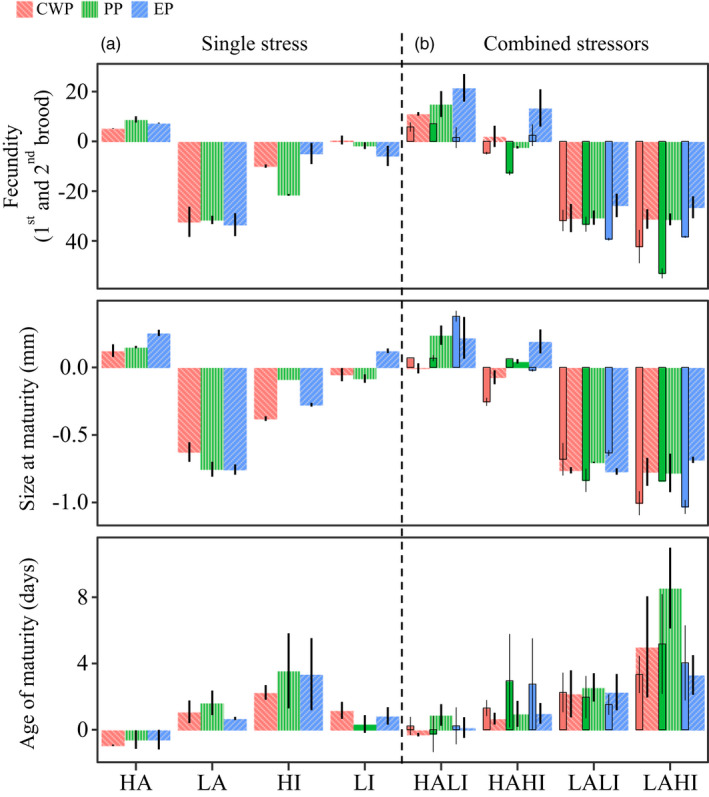
Effects of multistress combinations. Interactions between combinations of stressors are interpreted using an additive null model (Cote et al., 2016). Empirical observations from single (a) and multiple (b) stressors are shown per population and for three life‐history traits (fecundity, age at maturity, size at maturity). The predicted additive effects are shown in solid colors next to each observed population effect (dashed bars). A *t*‐test used to assess significant departure from a null model of additivity is in Table S2. The effects are synergistic when their combined effect is larger than the sum of the two individual stressors; antagonistic when their combined effect is smaller than the sum of the two individual stressors; and additive when their combined effect is the sum of the two individual stressors

Our previous analysis identified the evolutionary mechanisms underpinning fitness–trait changes in response to single and multiple stressors. To complement this analysis, we then quantified the effect of multiple stressors on the life‐history traits using an additive null model (Piggott et al., 2015). Across the three populations separated in time, the three life‐history traits and the four treatments in which pairs of stressors were tested; interactions were 44.4% synergistic, 41.7% additive, and 14% antagonistic (Figure 4, Table S2). The effect of combined stressors on fecundity was synergistic in 83% and antagonistic in 17% of the observations (Figure 4, Table S2). Combined‐stressor effects on size at maturity were synergistic in 41.7%, antagonistic in 33.3%, and additive in 25% of comparisons (Figure 4, Table S2). The effect of combined stressors on age at maturity was 83.3% additive, 8.3% synergistic, and 8.3% antagonistic.

**FIGURE 5:**
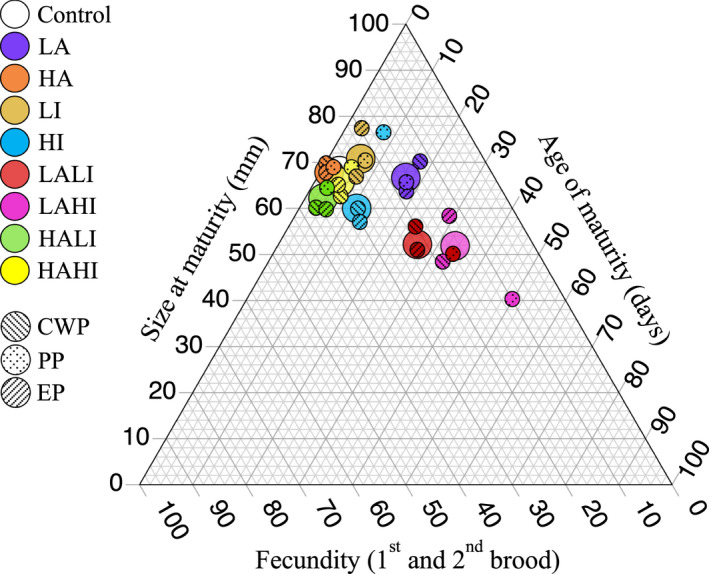
Trade‐off analysis. Ternary plot illustrating trilateral life‐history trade‐offs (fecundity, size at maturity, and age at maturity) in the single and multiple stressor exposures, calculated across the three *Daphnia* populations separated in time (CWP, PP, EP). Treatments are marked with different colors and the treatments are as follows: HA, high algae; HI, high insecticide; LA, low algae; LI, low insecticide. HAHI: high algae and high insecticide; HALI: high algae and low insecticide; LAHI: low algae and high insecticide; LALI: low algae and high insecticide. Populations are marked with different patterns

Regarding potential trade‐offs among traits, we observed that under control conditions *D*. *magna* displayed relatively short time to reach maturity, large body size, and high fecundity (Figure 5). The largest trade‐offs were observed when resource limitation (low algae, LA) was combined with insecticide treatment (both low‐LALI, and high‐LAHI). Under LALI and LAHI, the overall *Daphnia* fitness was lower than in other treatments, showing smaller size at maturity, delayed maturation, and lower fecundity. Conversely, high‐resource availability (high algae, HA) mitigated the adverse effect of high‐insecticide treatment, showing no clear departure of life‐history traits trade‐offs from control conditions (HAHI). The combination of high algae and low insecticide (HALI) showed a delay in maturity, which traded off with larger size at maturity and higher fecundity. Insecticide treatment in the presence of nonlimiting resources (animals fed *ad libitum*, HI) caused a shift in trade‐offs: HI and LI showed similar fecundity, but different size and age at maturity. HI exposed *Daphnia* had a delayed maturation as compared to LI exposed and control animals. LI induced large size at maturity but earlier maturation than the control (Figure 5). HA showed similar trade‐offs than control condition, whereas LA showed lower fecundity but similar size and age at maturity than HA. The trade‐offs induced by the stress combinations were generally similar across the temporal populations, except for PP in LALI and HI and EP in HI and LAHI, which were set apart from the other two populations (Figure 5).

## 4 DISCUSSION

Interactions between environmental stressors present one of the greatest uncertainties when predicting ecological changes. Yet, identifying multiple stressors impact on natural ecosystems and evolutionary mechanisms underpinning species response to multistress environments is paramount for the prioritization of conservation actions and mitigation interventions (Cote et al., 2016). The genetic background of spatially distributed populations can influence response to multiple stressors, leading to inconclusive evidence on the combined effects of environmental stressors (e.g., Sniegula et al., 2017).

Our study system enables us to quantify fitness trait evolution across multiple generations and environments without confounding factors linked to varying genetic background. By studying temporal population of *Daphnia* originating from the same genetic pool and mapping changes into known environmental shift in Lake Ring across 6 decades, we were able to identify and quantify the relative contribution of mechanisms of adaptation (plasticity, genetic adaptation, and evolution of plasticity) to complex environments. By quantifying fitness responses both to single stress and combinations of stressors, we show that single‐stress scenarios may not accurately capture mechanisms driving evolution if multiple stressors are in fact present (e.g., Stoks et al., 2016). Our study provides important insights into how historical exposure to multiple stressors can influence species fitness in modern environments.

We set out to address three main questions, which we discuss here in light of our results.

1. Does exposure to one stress result in higher tolerance to a second stressor?

Chemicals are a growing concern, especially the ones that are persistent in the environment (e.g., pesticides, pharmaceuticals; Choo et al., 2020). They impose augmented adverse effects when co‐occurring with other stressors because, for example, physical conditions (e.g., temperature) and nutrient levels can change their toxicokinetic rates of absorption, distribution, metabolism, or excretion (Choo et al., 2020). Moreover, chemicals have been shown to interact with organisms' physiology, reducing tolerance to other stressors (e.g., Højer et al., 2001; Noyes et al., 2009). In invertebrates, resource limitation has been shown to synergistically enhance chemical toxicity reducing organisms' fitness (Campero et al., 2008; Cuenca‐Cambronero et al., 2018; Jansen et al., 2015; Jansen, De Meester, et al., 2011). Our results show that the effect of the insecticide Carbaryl on *Daphnia* fitness is more severe when resource (algae) availability is limiting, whereas it is dampened by high‐resource availability. This may be explained by a buffering effect of the algae, which contribute to reduce chemicals in the environment by direct uptake or it can be explained by better coping mechanisms of the grazer (*Daphnia*) associated with allocation of resources. Our observations on life‐history trade‐offs support the latter. We observe the most severe trade‐offs in treatments involving the insecticide treatment (low and high concentrations), both when single‐stress and combined‐stress scenarios are considered. In treatments where resource limitation is combined with insecticide, fitness is lower than in control conditions and other exposures, showing smaller size at maturity, delayed maturation, and lower fecundity. Conversely, high‐resource availability combined with insecticide levels results in life‐history trade‐offs similar to control conditions. These results show that exposure to one stressor can alter tolerance to second stressors and that the interaction effects depend on the severity of either stressor.

- What mechanisms of adaptation (genetic evolution, plasticity, evolution of plasticity) enable long‐term evolution to multistress environments?

We studied adaptation mechanisms underpinning trait evolution in the three temporal populations of *Daphnia*. We complemented the analysis of overall trait variation in single and multistress scenarios with a targeted analysis of trait changes between the two historical transitions in Lake Ring, involving changes in biocides and resource (algae) availability. We found evidence for rapid evolutionary responses across the fitness‐linked life‐history traits. The evolutionary changes were explained by genetic evolution in fecundity, size at maturity, and mortality, as well as by plasticity in all traits. The evolutionary responses in the *Daphnia* populations across the two transitions studied were different (T1 → T2 and T2 → T3). In the transition between EP and PP (T1 → T2), characterized by significant changes in biocide levels but no changes in resource availability, evolution, evolution of plasticity, and plasticity all played an almost equal role in driving fitness responses. In the transition between PP and CWP (T2 → T3), in which both resource availability and biocides declined, plasticity played a dominant role in driving fitness responses.

The analysis of adaptation mechanisms in the lake transitions was complemented by the comparative analysis of single and multiple stressor scenarios on overall trait variation. This analysis revealed striking differences in the two scenarios. Theory predicts that plasticity more commonly occurs during the onset of a novel selection pressure (Crispo et al., 2010); this theory has been supported by empirical studies on single‐stress scenarios (Stoks et al., 2016). In the hypothetical single‐stress scenarios involving resource availability (HA, LA) in our study, the mechanisms underpinning trait changes were almost exclusively plastic, in agreement with theory predictions (Crispo et al., 2010; Hendry, 2015). However, the single scenarios involving changes in biocides (HI, LI), both from low to high concentrations and vice versa, do not align with theory predictions (Crispo et al., 2010; Hendry, 2015) and are in fact driven by an interaction of evolution, evolution of plasticity and plasticity. It is reasonable to expect that plasticity plays a more important role in the response to environmental stressors that vary seasonally and yearly (e.g., resource availability), enabling a rapid accommodation to environmental change. Similarly, it is expected that genetic evolution underpins responses to anthropogenic stressors, which may induce population bottleneck at their onset, and are more severe and rapid (Hendry, 2013). Taking a single‐stress scenarios perspective and ignoring the complexity of natural environments can lead to wrong estimates of the driving forces underpinning phenotypic evolution. This is important, because these traits can impact population persistence and evolutionary potential, which has implications for the conservation of populations.

- Is synergism more common than other interactions in Daphnia, supporting evidence from other population‐level studies?

Meta‐analyses on the interaction effect of multiple stressors on population fitness and community diversity show that the cumulative effects of multiple stressors are often worse than expected based on single‐stressor impacts, both in freshwater and in marine habitats (Crain et al., 2008; Darling & Cote, 2008; Holmstrup et al., 2010; Jackson et al., 2016; Jackson & Blois, 2015), with meta‐analyses reporting more than 50% synergistic effects on fitness when a chemical stressor is involved (Holmstrup et al., 2010). However, interaction between nonanthropogenic stressors (e.g., predator and nutrients) can lead to nonsignificant interactions despite the fact that they are expected to impact the same population or community trait (e.g., biomass; Borer et al., 2006).

In our study, we assessed the interaction effect of stressors relevant to the first and second transitions in the temporal populations of Lake Ring. Synergistic effects were most common across the three life‐history traits and exposures (44.4%), followed by additive effects (41.7%). Antagonistic effects were found in only 17% of observations. However, not all life‐history traits responded in the same way to the combination of stressors. Whereas the combined effect of stressors on size at maturity and fecundity was predominantly synergistic (in combination with some additive effects), combined effects for age at maturity were almost solely additive (83.3%). These results largely align with previous findings (Darling & Cote, 2008; Holmstrup et al., 2010; Jackson et al., 2016) but also show that conclusions on the effect of combined stressors may change with the fitness trait studied.

Stressor combinations resulted in different impact on fitness traits. We observed more severe adverse effects on traits and populations when resource limitation (low algae) interacted with insecticide, both at low and at high concentrations. Specifically, the overall fitness measured across age at maturity, size at maturity, and fecundity was lower than in any other stress combination and individual stress exposures. Conversely, high‐resource availability mitigated the effects of insecticide on fitness traits. It can, therefore, be expected that in the first transition in which biocide concentrations increased but resource availability remained high, the *Daphnia* population used compensatory mechanisms to cope with biocide pollution. Conversely, in the second transition both resource availability and biocides declined. According to our trade‐off analysis, both high and low concentrations of biocides combined with low‐resource availability impose stronger trade‐offs resulting in lower fecundity, delayed maturation, and smaller size at maturity. Our results also indicate that mortality is higher in presence of low‐resource availability combined with insecticide, both low and high. Based on these results, we expect the second transition to have had more severe impacts on the *Daphnia* population studied here. Importantly, the adverse effects of multiple stressors on *Daphnia* can have cascading effects on freshwater aquatic food webs, to which this species is central (Altshuler et al., 2011; Miner et al., 2012).

## 5 CONCLUSIONS

By studying responses to single and multiple stress scenarios in populations separated in time and originating from the same genetic pool, we were able to make inferences on the evolution of fitness traits in response to complex environments and on the role that historical exposure to environmental stress plays in this evolutionary response. Our results show that trait evolution in response to complex environments results from a combination of plastic and genetic mechanisms.

Single‐stress scenarios portray a different picture than multistress scenarios, showing that failing to account for complexity can result in a biased assessment of population response to changing environments. For example, the most severe impact on fitness was observed when resource limitation and biocides co‐occurred, causing significantly higher mortality, reduced fecundity and delay in maturation. However, the effect of biocides was somewhat dampened by higher resource availability, indicating that tolerance to one stress may be influenced by the severity of a second stressor. Considering the effect of biocides and of excess algae in isolation would have led to different evaluations of impact.

Only by quantifying the impact of multiple stressors, can we quantify species resilience in the face of future environmental change and prioritize mitigation interventions of environmental stressors causing significant adverse effects on biodiversity. Ecosystem managers are required to address and mitigate the impact of multiple stressors, yet the knowledge required to assess the impact of multiple stressors is still incomplete. Experimental studies like the one presented here help advance our understanding of single‐ and combined‐stressor effects, providing a better guide for conservation practices and prioritization of mitigation interventions.

## CONFLICT OF INTEREST

The authors declare no conflict of interest.

## Supporting information

Supplementary Material

## Data Availability

Data for this study are available at the Dryad Digital Repository: https://doi.org/10.5061/dryad.q83bk3jht
