# Supplementary material for: Evolutionary mechanisms underpinning fitness response to multiple stressors in Daphnia
Source: Evol Appl. 2021 Jun 10;14(10):2457–69. doi: 10.1111/eva.13258 (PMC8549616; doi:10.1111/eva.13258)
Supplement: Supplementary file 1 — Supplementary Material [file EVA-14-2457-s001.docx]

**Supporting information**

**Table S1. Univariate ANOVA per transition**

Univariate ANOVA supporting Figure 3 in the main text, testing the effect of environment (E), genetic evolution (G) and their interaction (G*E) on fecundity, size at maturity and age at maturity. These effects are calculated for the lake transitions EP → PP and PP → CWP using single stressors and combination of stressors (here representing the historical environments). Significant *P*-values are in bold. Populations are as follows: Eutrophic population - EP; Pesticide population - PP, Clear Water Population - CWP. High algae – HA, Low algae – LA, High insecticide – HI, low insecticide – LI, and their combinations.

|  |  | Fecundity (1st and 2nd brood) | | | | Size at maturity (mm) | | | | Age at maturity (dyas) | | | |
| --- | --- | --- | --- | --- | --- | --- | --- | --- | --- | --- | --- | --- | --- |
|  |  | Estimate | Chisq | Df | *p-value* | Estimate | Chisq | Df | *p-value* | Estimate | Chisq | Df | *p-value* |
| from EP to PP (HALI→HAHI) | G | -7.830 | 7.201 | 1 | ***0.014*** | 0.005 | 0.572 | 1 | 0.458 | 0.592 | 0.203 | 1 | 0.657 |
|  | E | -8.095 | 7.521 | 1 | ***0.013*** | -0.027 | 0.876 | 1 | 0.360 | 0.857 | 1.140 | 1 | 0.298 |
|  | G*E | -9.305 | 1.059 | 1 | 0.316 | -0.167 | 0.643 | 1 | 0.432 | -0.791 | 0.671 | 1 | 0.422 |
| from PP to CWP (HAHI→LALI) | G | 5.330 | 1.236 | 1 | 0.278 | -0.117 | 1.589 | 1 | 0.220 | -0.128 | 0.067 | 1 | 0.798 |
|  | E | -28.267 | 153.237 | 1 | ***0.000*** | -0.748 | 116.912 | 1 | ***0.000*** | 1.600 | 4.904 | 1 | ***0.038*** |
|  | G*E | -4.559 | 0.803 | 1 | 0.379 | 0.059 | 0.196 | 1 | 0.662 | -0.100 | 0.005 | 1 | 0.944 |
| from EP to PP (LI→HI) | G | 2.829 | 1.051 | 1 | 0.322 | -0.221 | 3.054 | 1 | 0.101 | -0.660 | 0.222 | 1 | 0.645 |
|  | E | 0.945 | 1.771 | 1 | 0.203 | -0.400 | 16.832 | 1 | ***0.001*** | 2.523 | 9.443 | 1 | ***0.008*** |
|  | G*E | -20.730 | 5.045 | 1 | ***0.040*** | 0.395 | 4.677 | 1 | ***0.047*** | 0.690 | 0.133 | 1 | 0.721 |
| from PP to CWP (HA→LA) | G | 12.466 | 3.091 | 1 | 0.101 | -0.291 | 0.240 | 1 | 0.633 | -1.145 | 0.164 | 1 | 0.693 |
|  | E | 19.785 | 14.904 | 1 | ***0.002*** | 0.004 | 10.398 | 1 | ***0.007*** | -3.213 | 6.650 | 1 | ***0.023*** |
|  | G*E | -9.287 | 1.546 | 1 | 0.234 | 0.321 | 4.087 | 1 | 0.064 | 2.130 | 2.003 | 1 | 0.181 |
| from PP to CWP (HI→LI) | G | -2.659 | 0.596 | 1 | 0.445 | -0.025 | 1.117 | 1 | 0.297 | -0.182 | 0.557 | 1 | 0.460 |
|  | E | -40.403 | 620.243 | 1 | ***0.000*** | -0.904 | 302.782 | 1 | ***0.000*** | 2.222 | 32.659 | 1 | ***0.000*** |
|  | G*E | 2.819 | 0.792 | 1 | 0.379 | 0.154 | 2.623 | 1 | 0.113 | -0.197 | 0.069 | 1 | 0.794 |

**Table S2. Synergistic, antagonistic and additive effects of combined stressors**

T-test results of mean life history trait values (effect size) per population and treatment testing their departure from a null model of additivity in the analysis of combined effect of stressors (synergistic, antagonistic and additive). Treatments are as Table S1.

|  |  | Fecundity | | | Size at maturity | | | Age at maturity | | |
| --- | --- | --- | --- | --- | --- | --- | --- | --- | --- | --- |
| Treatment | Pop | t-value | P-value | Effect | t-value | P-value | Effect | t-value | P-value | Effect |
| HALI | CWP | 132.04 | ***0.000*** | Syn | 37.45 | ***0.000*** | An | 8.72 | ***0.009*** | An |
|  | PP | 14.51 | ***0.003*** | Syn | 43.82 | ***0.000*** | An | 5.05 | 0.046 | Add |
|  | EP | 63.58 | ***0.000*** | Syn | 8.52 | ***0.012*** | Syn | 0.05 | 0.827 | Add |
| HAHI | CWP | 22.33 | ***0.000*** | Syn | 75.05 | ***0.000*** | Syn | 7.83 | ***0.015*** | Syn |
|  | PP | 530.72 | ***0.000*** | Syn | 2.30 | 0.168 | Add | 2.31 | 0.163 | Add |
|  | EP | 25.33 | ***0.000*** | Syn | 44.61 | ***0.000*** | Syn | 2.74 | 0.122 | Add |
| LALI | CWP | 0.18 | 0.676 | Add | 4.08 | 0.060 | Add | 0.02 | 0.894 | Add |
|  | PP | 2.59 | 0.136 | Add | 12.80 | ***0.004*** | An | 0.80 | 0.392 | Add |
|  | EP | 75.42 | ***0.000*** | Syn | 176.39 | ***0.000*** | An | 3.08 | 0.101 | Add |
| LAHI | CWP | 14.69 | ***0.002*** | Syn | 16.58 | ***0.002*** | Syn | 2.01 | 0.187 | Add |
|  | PP | 209.86 | ***0.000*** | Syn | 2.54 | 0.172 | Add | 2.79 | 0.139 | Add |
|  | EP | 69.17 | ***0.000*** | Syn | 214.73 | ***0.000*** | Syn | 0.35 | 0.565 | Add |

**Table S3. Post-hoc analysis**

Post-hoc analysis supporting univariate three-way ANOVA in Table 2 on the four life history traits measured in this study. Pairwise comparisons between populations (EP, PP and CWP) are shown for single stressors (high -HA, and low-LA; high – HI and low - LI) and stressor combinations (HAHI, HALI, LAHI. And LALI.

|  |  | Fecundity | Size at maturity | Age at maturity | Mortality |
| --- | --- | --- | --- | --- | --- |
|  | Contrast | *p-value* | *p-value* | *p-value* | *p-value* |
| Population | PP-CWP | 1.000 | 0.140 | 0.160 | 0.413 |
|  | EP-CWP | ***0.005*** | ***0.020*** | 1.000 | 0.265 |
|  | EP-PP | ***0.003*** | 1.000 | 0.259 | ***0.008*** |
| Algae | LA-HA | ***0.000*** | ***0.000*** | ***0.000*** | ***0.008*** |
| Insecticide | LI-HI | ***0.001*** | 0.364 | ***0.000*** | 0.069 |
| Pop * Algae | PP:HA-CWP:HA | 1.000 | 0.406 | 1.000 | 1.000 |
|  | EP:HA-CWP:HA | ***0.007*** | ***0.011*** | 1.000 | 1.000 |
|  | CWP:LA-CWP:HA | ***0.000*** | ***0.000*** | ***0.000*** | 0.811 |
|  | PP:LA-CWP:HA | ***0.000*** | ***0.000*** | ***0.000*** | 1.000 |
|  | EP:LA-CWP:HA | ***0.000*** | ***0.000*** | ***0.004*** | 0.056 |
|  | EP:HA-PP:HA | ***0.007*** | 1.000 | 1.000 | 0.801 |
|  | CWP:LA-PP:HA | ***0.000*** | ***0.000*** | ***0.033*** | 0.181 |
|  | PP:LA-PP:HA | ***0.000*** | ***0.000*** | ***0.000*** | 1.000 |
|  | EP:LA-PP:HA | ***0.000*** | ***0.000*** | 0.160 | ***0.010*** |
|  | CWP:LA-EP:HA | ***0.000*** | ***0.000*** | ***0.006*** | 1.000 |
|  | PP:LA-EP:HA | ***0.000*** | ***0.000*** | ***0.000*** | 1.000 |
|  | EP:LA-EP:HA | ***0.000*** | ***0.000*** | ***0.041*** | 1.000 |
|  | PP:LA-CWP:LA | 1.000 | 1.000 | 0.601 | 1.000 |
|  | EP:LA-CWP:LA | 1.000 | 1.000 | 1.000 | 1.000 |
|  | EP:LA-PP:LA | 1.000 | 1.000 | 0.273 | 0.311 |
| Pop * Instct. | PP:HI-CWP:HI | 1.000 | 1.000 | 1.000 | 1.000 |
|  | EP:HI-CWP:HI | 0.320 | 0.369 | 1.000 | 1.000 |
|  | CWP:LI-CWP:HI | 1.000 | 1.000 | 0.162 | 0.793 |
|  | PP:LI-CWP:HI | 0.728 | 0.279 | 1.000 | 0.227 |
|  | EP:LI-CWP:HI | ***0.003*** | 0.619 | 0.621 | 1.000 |
|  | EP:HI-PP:HI | ***0.033*** | 1.000 | 0.389 | 1.000 |
|  | CWP:LI-PP:HI | 0.107 | 1.000 | ***0.001*** | 1.000 |
|  | PP:LI-PP:HI | 0.081 | 1.000 | ***0.042*** | 1.000 |
|  | EP:LI-PP:HI | ***0.000*** | 1.000 | ***0.009*** | 1.000 |
|  | CWP:LI-EP:HI | 1.000 | 0.779 | 0.972 | 0.368 |
|  | PP:LI-EP:HI | 1.000 | 1.000 | 1.000 | 0.102 |
|  | EP:LI-EP:HI | 1.000 | 1.000 | 1.000 | 1.000 |
|  | PP:LI-CWP:LI | 1.000 | 0.583 | 1.000 | 1.000 |
|  | EP:LI-CWP:LI | 0.210 | 1.000 | 1.000 | 0.650 |
|  | EP:LI-PP:LI | 0.837 | 1.000 | 1.000 | 0.188 |
| Alage*Insect. | LAHI-HAHI | ***0.000*** | ***0.000*** | ***0.000*** | 0.307 |
|  | HALI-HAHI | ***0.000*** | 1.000 | 1.000 | 1.000 |
|  | LALI-HAHI | ***0.000*** | ***0.000*** | 0.057 | 1.000 |
|  | HALI-LAHI | ***0.000*** | ***0.000*** | ***0.000*** | ***0.011*** |
|  | LALI-LAHI | 1.000 | 1.000 | ***0.000*** | 1.000 |
|  | LALI-HALI | ***0.000*** | ***0.000*** | ***0.001*** | 0.396 |

|  |  | Fecundity | Size at  maturity | Age at  maturity | Mortality |
| --- | --- | --- | --- | --- | --- |
|  | Contrast | *p-value* | *p-value* | *p-value* | *p-value* |
| Pop*Algae*Ins. | PP:HAHI-CWP:HAHI | 1.000 | 1.000 | 1.000 | 1.000 |
|  | EP:HAHI-CWP:HAHI | 0.563 | 0.639 | 1.000 | 1.000 |
|  | CWP:LAHI-CWP:HAHI | ***0.000*** | ***0.000*** | ***0.015*** | 1.000 |
|  | PP:LAHI-CWP:HAHI | ***0.000*** | ***0.002*** | ***0.000*** | 1.000 |
|  | EP:LAHI-CWP:HAHI | ***0.000*** | ***0.000*** | 1.000 | 1.000 |
|  | CWP:HALI-CWP:HA:HI | 1.000 | 1.000 | 1.000 | 1.000 |
|  | PP:HALI-CWP:HA:HI | 0.477 | 0.573 | 1.000 | 1.000 |
|  | EP:HALI-CWP:HA:HI | ***0.001*** | 0.319 | 1.000 | 1.000 |
|  | CWP:LALI-CWP:HAHI | ***0.000*** | ***0.000*** | 1.000 | 1.000 |
|  | PP:LALI-CWP:HAHI | ***0.000*** | ***0.000*** | 1.000 | 1.000 |
|  | EP:LALI-CWP:HAHI | ***0.000*** | ***0.000*** | 1.000 | 1.000 |
|  | EP:HAHI-PP:HAHI | ***0.039*** | 1.000 | 1.000 | 1.000 |
|  | CWP:LAHI-PP:HAHI | ***0.000*** | ***0.000*** | ***0.036*** | 0.851 |
|  | PP:LAHI-PP:HAHI | ***0.000*** | ***0.000*** | ***0.000*** | 1.000 |
|  | EP:LAHI-PP:HAHI | ***0.001*** | ***0.000*** | 1.000 | 1.000 |
|  | CWP:HALI-PP:HA:HI | 0.105 | 1.000 | 1.000 | 1.000 |
|  | PP:HALI-PP:HA:HI | ***0.032*** | 1.000 | 1.000 | 1.000 |
|  | EP:HALI-PP:HA:HI | ***0.000*** | 1.000 | 1.000 | 1.000 |
|  | CWP:LALI-PP:HAHI | ***0.000*** | ***0.000*** | 1.000 | 1.000 |
|  | PP:LALI-PP:HAHI | ***0.000*** | ***0.000*** | 1.000 | 1.000 |
|  | EP:LALI-PP:HAHI | ***0.001*** | ***0.000*** | 1.000 | 0.432 |
|  | CWP:LAHI-EP:HAHI | ***0.000*** | ***0.000*** | ***0.028*** | 1.000 |
|  | PP:LAHI-EP:HAHI | ***0.000*** | ***0.000*** | ***0.000*** | 1.000 |
|  | EP:LAHI-EP:HAHI | ***0.000*** | ***0.000*** | 1.000 | 1.000 |
|  | CWP:HALI-EP:HA:HI | 1.000 | 1.000 | 1.000 | 1.000 |
|  | PP:HALI-EP:HA:HI | 1.000 | 1.000 | 1.000 | 1.000 |
|  | EP:HALI-EP:HA:HI | 1.000 | 1.000 | 1.000 | 1.000 |
|  | CWP:LALI-EP:HAHI | ***0.000*** | ***0.000*** | 1.000 | 1.000 |
|  | PP:LALI-EP:HAHI | ***0.000*** | ***0.000*** | 1.000 | 1.000 |
|  | EP:LALI-EP:HAHI | ***0.000*** | ***0.000*** | 1.000 | 1.000 |
|  | PP:LAHI-CWP:LAHI | 1.000 | 1.000 | 0.790 | 1.000 |
|  | EP:LAHI-CWP:LAHI | 1.000 | 1.000 | 1.000 | 1.000 |
|  | CWP:HALI-CWP:LAHI | ***0.000*** | ***0.000*** | ***0.000*** | 0.439 |
|  | PP:HALI-CWP:LAHI | ***0.000*** | ***0.000*** | ***0.018*** | 1.000 |
|  | EP:HALI-CWP:LAHI | ***0.000*** | ***0.000*** | ***0.002*** | 1.000 |
|  | CWP:LALI-CWP:LAHI | 1.000 | 1.000 | 0.520 | 1.000 |
|  | PP:LALI-CWP:LAHI | 1.000 | 1.000 | 1.000 | 1.000 |
|  | EP:LALI-CWP:LAHI | 1.000 | 1.000 | 0.924 | 1.000 |
|  | EP:LAHI-PP:LAHI | 1.000 | 1.000 | ***0.017*** | 1.000 |
|  | CWP:HALI-PP:LAHI | ***0.000*** | ***0.000*** | ***0.000*** | 1.000 |
|  | PP:HALI-PP:LAHI | ***0.000*** | ***0.000*** | ***0.000*** | 1.000 |

|  |  | Fecundity | Size at maturity | Age at maturity | Mortality |
| --- | --- | --- | --- | --- | --- |
|  | Contrast | *p-value* | *p-value* | *p-value* | *p-value* |
| Pop*Algae*Ins. (continuation) | EP:HALI-PP:LAHI | ***0.000*** | ***0.000*** | ***0.000*** | 1.000 |
|  | CWP:LALI-PP:LAHI | 1.000 | 1.000 | ***0.000*** | 1.000 |
|  | PP:LALI-PP:LAHI | 1.000 | 1.000 | ***0.001*** | 1.000 |
|  | EP:LALI-PP:LAHI | 1.000 | 1.000 | ***0.000*** | 1.000 |
|  | CWP:HALI-EP:LAHI | ***0.000*** | ***0.000*** | ***0.048*** | 0.869 |
|  | PP:HALI-EP:LAHI | ***0.000*** | ***0.000*** | 1.000 | 1.000 |
|  | EP:HALI-EP:LAHI | ***0.000*** | ***0.000*** | 0.292 | 1.000 |
|  | CWP:LALI-EP:LAHI | 1.000 | 1.000 | 1.000 | 1.000 |
|  | PP:LALI-EP:LAHI | 1.000 | 1.000 | 1.000 | 1.000 |
|  | EP:LALI-EP:LAHI | 1.000 | 1.000 | 1.000 | 1.000 |
|  | PP:HALI-CWP:HALI | 1.000 | 1.000 | 1.000 | 1.000 |
|  | EP:HALI-CWP:HALI | 0.510 | 1.000 | 1.000 | 1.000 |
|  | CWP:LALI-CWP:HALI | ***0.000*** | ***0.000*** | 0.178 | 1.000 |
|  | PP:LALI-CWP:HALI | ***0.000*** | ***0.000*** | 0.374 | 1.000 |
|  | EP:LALI-CWP:HALI | ***0.000*** | ***0.000*** | 0.223 | 0.211 |
|  | EP:HALI-PP:HALI | 1.000 | 1.000 | 1.000 | 1.000 |
|  | CWP:LALI-PP:HALI | ***0.000*** | ***0.000*** | 1.000 | 1.000 |
|  | PP:LALI-PP:HALI | ***0.000*** | ***0.000*** | 1.000 | 1.000 |
|  | EP:LALI-PP:HALI | ***0.000*** | ***0.000*** | 1.000 | 0.659 |
|  | CWP:LALI-EP:HALI | ***0.000*** | ***0.000*** | 1.000 | 1.000 |
|  | PP:LALI-EP:HALI | ***0.000*** | ***0.000*** | 1.000 | 1.000 |
|  | EP:LALI-EP:HALI | ***0.000*** | ***0.000*** | 1.000 | 1.000 |
|  | PP:LALI-CWP:LALI | 1.000 | 1.000 | 1.000 | 1.000 |
|  | EP:LALI-CWP:LALI | 1.000 | 1.000 | 1.000 | 1.000 |
|  | EP:LALI-PP:LALI | 1.000 | 1.000 | 1.000 | 0.659 |

**Figure S1.**

A) Schematic methodological illustration showing the univariate reaction norms component for a certain phenotypic trait with mean $\bar{y}$ in two different environments at time 1 (T1) and time 2 (T2). In this figure, total trait change (dashed line) can be divided in three components: plasticity (here given as the different in trait value between T1 and T2 for a certain population, e.g. EP to PP), genetic evolution (genetic difference between two (sub)population, e.g. EP to PP) and evolution of plasticity (difference in trait slope between T1 and T2). Figure adapted from Govaert et al. (2016). B) Historical environment over the past 80 years in Lake Ring. T1 was characterized by high algae and low insecticide (HALI), T2 was characterized by high algae and insecticide (HAHI), and time 3 (T3) was characterized by low concentration of algae and insecticide (LALI).
